# Epidemiological characteristics of the SARS-CoV-2 Theta variant (P.3) in the Central Visayas region, Philippines, 30 October 2020–16 February 2021

**DOI:** 10.5365/wpsar.2022.13.1.883

**Published:** 2022-02-09

**Authors:** Nel Jason L. Haw, Eugenia Mercedes R. Cañal, Juanito Zuasula, Mary Jean Loreche, Jaime Bernadas

**Affiliations:** a Health Sciences Program, Ateneo de Manila University, Quezon City, Philippines.; b Department of Epidemiology, Johns Hopkins Bloomberg School of Public Health, Baltimore, MD, United States of America.; c Center for Health and Development, Region 7 (Central Visayas Region), Department of Health, Cebu City, Philippines.

The Theta variant of severe acute respiratory syndrome coronavirus 2 (SARS-CoV-2) was first reported in the Central Visayas region, Philippines, in January 2021. The World Health Organization (WHO) designated the Theta variant as a variant of interest on 24 March 2021, (*1*) given that it contains the mutations E484K, which has been associated with antigenic escape; N501Y, associated with increased transmissibility; and other mutations of concern found in existing variants of concern (Tablizo FA, Kim KM, Lapid CM, Castro MJR, Yangzon MSL, Maralit BA, et al. Philippine Genome Center, unpublished data, 2021). This report is the first to describe the epidemiological profile of this variant.

Surveillance for pathogens in the Philippines is coordinated at the national level by the Department of Health Epidemiology Bureau and at the regional level by Regional Epidemiology and Surveillance Units (Regional Units); surveillance at the local level is conducted by City and Municipal Epidemiology and Surveillance Units. Regional Units identify candidates for sequencing among SARS-CoV-2 specimens that are positive by nucleic acid amplification test and send these samples to the Philippine Genome Center for whole-genome sequencing (WGS). The criteria for sample selection established by the Central Visayas Regional Unit include specimens from cases with known travel history from countries with variants of concern or interest, specimens that are part of large clusters of positive coronavirus 2019 (COVID-19) cases and specimens that have a volume > 500 µL and a cycle threshold value < 30 (Central Visayas Center for Health Development, unpublished guidance: collection and testing guidelines for genomic sequencing, 2021). To meet the national capacity for WGS, which is about 750 samples weekly, (*2*) the Epidemiology Bureau further narrows down samples for WGS.

As of 10 June 2021, 950 samples, one sample per case, had been sent for WGS from the Central Visayas region, and 321 (33.8%) had been sequenced. The samples were collected from 30 October 2020 to 16 February 2021. The WGS results were provided to the Central Visayas Regional Unit between 12 February and 12 March 2021.

Cases were followed up for 60 days post-diagnosis to determine whether they resolved through clinical recovery or death; clinical recovery was defined as the date when a case was discharged from a hospital or isolation facility following SARS-CoV-2 infection. Outcomes were ascertained based on standard discharge and recovery criteria as reported by City and Municipal Surveillance Units or local health facilities. (*3*)

Cases were excluded from the analysis if they had any of the four variants of concern identified as of June 2021 – that is, Alpha, Beta, Gamma or Delta – as these are known to be associated with higher transmissibility and mortality. Some specimens were from cases in the same outbreak cluster or shared the same home address and date of specimen collection; therefore, all statistical analyses considered clustering at this level. The risk ratio (RR) for COVID-19 hospitalization was calculated using a log-binomial generalized estimating equation with an independent working correlation structure and robust sandwich variance estimator. (*4*) Median recovery times were calculated from Kaplan–Meier survival curves. The hazard ratios (HRs) for recovery were estimated using a shared frailty model, which is an extension of the Cox proportional hazards model that accounts for clustering, and the proportional hazards assumption was checked using Schoenfeld residuals. (*4*) For both RR and HR, the models were adjusted for morbidity, week of specimen collection of first positive test, age, sex and province. Finally, because contact tracing is done by City and Municipal Surveillance Units, two major cities provided a copy of their database to the Regional Unit. For cases with the Theta variant in those cities, close contacts were counted and cross-referenced with the Regional Unit’s confirmed COVID-19 line list to determine which close contacts had tested positive. The secondary attack rate was calculated as the number of positive close contacts divided by the total number of close contacts. All analyses were conducted using Stata version 17 (StataCorp, College Station, TX, USA).

Of the 321 cases with WGS results, four had the variant of concern and were excluded; thus, the final sample size was 317 cases, comprising 2.6% of the 12 136 cases confirmed by nucleic acid amplification tests during the specimen collection period. The Theta variant was detected in 68 (21.5%) of the samples. For all cases, most specimens (200/317; 63.1%) and almost all specimens from Theta variant cases (64/68; 94.1%) were collected during the first 2 weeks of February  (**Table 1**). Additionally, most specimens were collected from cases in Cebu province, including from the independent cities of Cebu City, Lapu-Lapu City and Mandaue City (191 [60.3%] of specimens overall and 48/68 [70.6%] among Theta variant cases). The median age of all cases was 33 years (interquartile range [IQR]: 21–46), and 142 (44.8%) cases occurred among women. The age and sex distributions were similar between cases with the Theta and non-Theta variants (**Table 1**).

**Table 1:** Comparison of Theta variant and non-Theta variant SARS-CoV-2 cases in the Central Visayas region, Philippines, 30 October 2020–16 February 2021

Characteristic	Severe acute respiratory syndrome coronavirus 2		
-	All^a^ (*n* = 317)	Theta variant (*n* = 68)	Non-Theta variant (*n* = 249)
Female, *n*(%)	142 (44.8)	30 (44.1)	112 (45.0)
Age (years), median (IQR)	33 (21–46)	34.5 (20.5–42)	33 (21–49)
Age group (years), *n*(%)			
0–17	69 (21.8)	15 (22.1)	54 (21.7)
18–44	161 (50.8)	39 (57.3)	122 (49.0)
45–60	53 (16.7)	8 (11.8)	45 (18.1)
> 60	34 (10.7)	6 (8.8)	28 (11.2)
In Cebu province, *n*(%)^b^	191 (60.3)	48 (70.6)	143 (57.4)
Specimens collected during the first 2 weeks of February 2021, *n*(%)^c^	200 (63.1)	64 (94.1)	136 (54.6)

IQR: interquartile range.

^a^ Excludes four sequenced samples with variants of concern.

^b^ A full provincial breakdown is not available due to small sample sizes from some provinces (< 5 cases); counts from Cebu province include the independent cities of Cebu City, Lapu-Lapu City and Mandaue City.

^c^ Other dates of specimen collection are not available due to small sample sizes in some months (< 5 cases).

Hospitalization was required for 18 of the 68 cases with the Theta variant (26.5%) compared with 77 of the 249 non-Theta variant cases (30.9%) (data not shown). The risk for hospitalization was not significantly associated with having the Theta variant (crude RR: 0.86; 95% confidence interval [CI]: 0.48–1.51; adjusted RR: 0.76; 95% CI: 0.40–1.47) (data not shown). The median time to recovery was similar, at 17 days, and the HR for recovery also was not associated with the Theta variant (crude HR: 1.00; 95% CI: 0.70–1.44; adjusted HR: 0.92; 95% CI: 0.65–1.30) (data not shown). Three cases died, none of whom had the Theta variant.

Nineteen clusters were identified from two major cities, with the index case having the Theta variant. The median number of close contacts was 8 (IQR: 5–13). There were 182 close contacts, 81 of whom tested positive. Therefore, the overall secondary attack rate for cases infected with the Theta variant was 44.5%.

These preliminary results, showing that the Theta variant did not seem to be associated with more severe disease, were similar to findings on the Iota variant (B.1.526) in New York, which also contained the E484K mutation. (*5*) Additionally, while the secondary attack rate was more than twice as high as the 16.6% previously reported, (*6*) the number of clusters examined was too few to draw any definitive conclusions. Since March 2021, most of the country’s biosurveillance capacity has been dedicated to testing samples from the National Capital Region and from repatriated Filipinos. (*7*) Further investments to expand biosurveillance capacity, as well as to facilitate timelier linkages of genomic, epidemiological and clinical data, may be needed to further understand the epidemiological profile of the Theta variant. Nonetheless, our results can contribute to the evidence base used by WHO and countries affected by the Theta variant to clarify its status as a variant of interest.
